# Nonalcoholic fatty liver disease and obesity: An Obesity Medicine Association (OMA) Clinical Practice Statement (CPS) 2022

**DOI:** 10.1016/j.obpill.2022.100027

**Published:** 2022-07-08

**Authors:** Sara Karjoo, Anthony Auriemma, Teresa Fraker, Harold Edward Bays

**Affiliations:** a University of South Florida, 12901 Bruce B Downs Blvd, Tampa, FL, 33612, USA; b Florida State University, 1115 W Call St., Tallahassee, FL, 32304, USA; c Johns Hopkins School of Medicine, 733 N Broadway, Baltimore, MD, 21205, USA; d Ascension Illinois Medical Group Weight Loss Solutions, 25 E Schaumburg Rd, Suite 101, Schaumburg, IL, 60194, USA; e Obesity Medicine Association, 7173 South Havana Street #600-130, Centennial, CO, 80112, USA; f Louisville Metabolic and Atherosclerosis Research Center, 3288 Illinois Avenue, 40213, USA; g University of Louisville School of Medicine, 500 S Preston St, Louisville, KY, 40202, USA

**Keywords:** Adiposopathy, Clinical practice statement, Nonalcoholic fatty liver disease, Obesity, Pre-obesity

## Abstract

**Background:**

This Obesity Medicine Association (OMA) Clinical Practice Statement (CPS) provides clinicians an overview of nonalcoholic fatty liver disease (NAFLD), potential progression to nonalcoholic steatohepatitis (NASH), and their application to obesity.

**Methods:**

The scientific information for this CPS is based upon published scientific citations, clinical perspectives of OMA authors, and peer review by the Obesity Medicine Association leadership.

**Results:**

Topics of this CPS include the prevalence of NAFLD and NASH, the prevalence of NAFLD and NASH among patients with obesity, as well as NAFLD and NASH definitions, diagnosis, imaging, pathophysiology, differential diagnosis, role of high fructose corn syrup and other simple sugars, and treatment (e.g., nutrition, physical activity, medications).

**Conclusions:**

This Obesity Medicine Association (OMA) Clinical Practice Statement (CPS) regarding NAFLD and obesity is one of a series of OMA CPSs designed to assist clinicians in the care of patients with the disease of obesity. Patients with obesity are at increased risk for NAFLD and NASH. Patients may benefit when clinicians who manage obesity understand the etiology, diagnosis, and optimal treatment of NAFLD with a goal to prevent NASH.

## 1 Introduction

Beginning in 2013, the Obesity Medicine Association (OMA) created and maintained an online Adult “Obesity Algorithm” (i.e., educational slides and eBook) that underwent yearly updates by OMA authors and was reviewed and approved annually by the OMA Board of Trustees [1]. This was followed by a similar Pediatric “Obesity Algorithm” with updates approximately every two years by OMA authors. This OMA Clinical Practice Statement (CPS) regarding nonalcoholic fatty liver disease was derived from the 2021 OMA Adult Obesity Algorithm and is one of a series of OMA CPSs designed to assist clinicians in the care of patients with the disease of obesity.

### 1.1 Obesity and nonalcoholic fatty liver disease (NAFLD)

Nonalcoholic fatty liver disease (NAFLD) encompasses a spectrum of fatty liver diseases. NAFLD is the most common cause of chronic liver disease, affecting approximately 25% of adults [2]. Worldwide, the prevalence of NAFLD is highest in South Asia, Middle East, Mexico, as well as Central and South America (≥30%). The prevalence NAFLD is reportedly more moderate in the United States, Europe, and East Asia (23–27%). The prevalence of NAFLD is lowest in Africa (14%) [3].

**Fig. 1:**
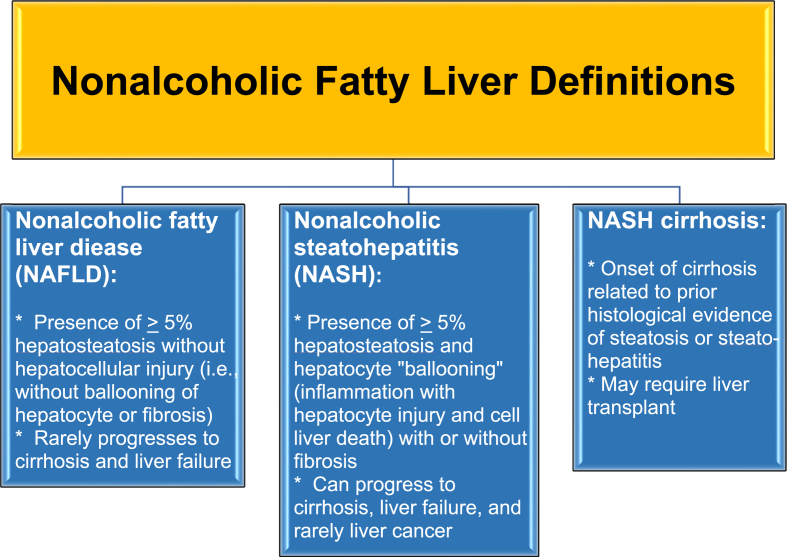
NAFLD Definitions. Shown are the definitions of NAFLD, nonalcoholic fatty liver, nonalcoholic steatohepatitis (NASH), and NASH cirrhosis [24]. NAFLD encompasses the spectrum of fatty liver not related to alcohol consumption: fatty liver, hepatosteatitis, and cirrhosis.

In the United States, the reported prevalence of NAFLD by race can vary considerably, depending on the report, population, and diagnostic methodology [4]. Hispanic individuals are sometimes reported to have the highest prevalence rates, followed by White and Black individuals (21%, 12.5%, and 11.6% respectively) [3]. As previously noted, the prevalence of NAFLD among Asians is approximately 30% [5], highlighting the importance of recognizing the unique nutritional considerations from those of Asian descent [6], and the unique adipose tissue and cardiovascular risk pathophysiology commonly found among those from South Asian [7]. Irrespective of race, NAFLD is especially prevalent in individuals with obesity, and more than two thirds of patients diagnosed with NAFLD have obesity [8]. In recognition that NAFLD is often associated with metabolic dysfunction, some have suggested the alternative term “metabolic dysfunction associated fatty liver disease” (MAFLD) [9,10]. All that said, 10–20% of patients with NAFLD are lean by body mass index criteria [3]. Table 1 shows ten takeaway messages regarding obesity and nonalcoholic fatty liver disease. Fig. 1 displays fatty liver definitions.

**Table 1:** Ten takeaway messages: Obesity and nonalcoholic fatty liver disease.

1.	Non-alcoholic fatty liver disease (NAFLD) includes a spectrum of fatty liver diseases not due to alcohol intake (i.e., nonalcoholic fatty liver, nonalcoholic steatohepatitis (NASH), advanced fibrosis, cirrhosis, and hepatocellular carcinoma). NAFLD is the most common cause of chronic liver disease (>25% of all adults) [2,3,11]. Over 1/2 to 2/3 of patients with NAFLD have obesity and over ¾ of patients with NASH have obesity [12]. Among patients with NAFLD, 10–25% may have or develop NASH [3,13].
2.	NAFLD is a risk factor for cardiovascular disease [3,8]. Development of NASH is 2–3 times higher in patients with obesity and/or type 2 diabetes mellitus [14]. The prevalence of NASH in patients with obesity is 30%, while the prevalence of NASH in patients with type 2 diabetes mellitus ranges between 30% to over 50% [3,14].
3.	Hepatosteatosis or fatty liver is defined as ≥ 5% hepatic fat; NASH is the presence of ≥5% hepatic fat with inflammation and hepatocyte injury with or without fibrosis [[15], [16], [17]].
4.	After a 20-year follow-up, the risk of cirrhosis with hepatosteatosis is 0–4%. After a 9-year follow-up, the risk of cirrhosis with NASH may be 25% [18].
5.	NASH is an important cause of end stage liver disease, hepatocellular carcinoma, and is a leading indication for liver transplant (secondary to hepatitis C) [3,14,19].
6.	While some drugs are suggested to improve NASH, no drug has an approved indication to treat NASH [8].
7.	Simple screening for potential hepatosteatosis includes otherwise unexplained elevation in alanine transaminase (ALT) [often accompanied by elevated aspartate transaminase (AST)] especially in patients with obesity, type 2 diabetes mellitus [8], metabolic syndrome, or high triglyceride levels [3].
8.	Among the safest and most reliable imaging tests for fatty liver include transient elastography and magnetic resonance imaging proton density fat fraction (MRI-PDFF) or MR spectroscopy (MRS) [20].
9.	The adiposopathic consequence of obesity and use of some medications often lead to NAFLD and may contribute to insulin resistance, type 2 diabetes mellitus, and dyslipidemia (e.g., hypertriglyceridemia) [3]. Other NAFLD etiologies include genetic predisposition, autoimmune processes, medications, environmental exposure, and infectious diseases that can cause progressive fatty liver disease in patients with obesity [21,22].
10.	Management of NAFLD includes treatment of secondary causes, appropriate nutrition and physical activity, potential administration of peroxisome proliferator activated receptor gamma agonists or glucagon-like protein-1 receptors (i.e., the effects of metformin on NAFLD are inconclusive) [[23], [24], [25]], and bariatric surgery [26].

Abbreviations: ALT = alanine transaminase; AST = aspartate transaminase; MRI-PDFF = magnetic resonance imaging proton density fat fraction; MRS = magnetic resonance spectroscopy; NAFLD = nonalcoholic fatty liver disease; NASH = nonalcoholic steatohepatitis.

### 1.2 NAFLD diagnosis: simple blood screening tests

Hepatosteatosis (i.e., a condition of increased liver fat) typically results in elevated liver transaminases such as alanine transaminase (ALT) and aspartate transaminase (AST), with normal bilirubin. Increase in ALT is sometimes described as more specific for NAFLD. AST:ALT ratio of >1.5 may be more consistent with alcoholic liver disease; AST > ALT is associated with increased hepatic fibrosis [23,27,28]. The AST-to-platelet ratio is also sometimes used to evaluate NAFLD [14]. That said, some reports suggest that 25% of NAFLD patients and 19% of NASH patients have normal AST blood levels [29].

### 1.3 NAFLD diagnosis: imaging tests

Liver biopsy is the definitive test for diagnosis of NAFLD and NASH. Liver biopsies are currently required by the Food and Drug Administration as part of the development program for drugs approved to treat NAFLD and NASH, with no drugs yet approved for these indications [24,30,31]. The degree of liver steatosis can be graded based upon the histologic percent of fat in hepatocytes:

• Grade 0: <5%
• Grade 1: 5–33%
• Grade 2: 33–66%
• Grade 3: >66%

Beyond liver biopsy, and within the scope of both clinical research and clinical practice, NAFLD is often diagnosed by non-invasive hepatic imaging. Particularly in patients with obesity, hyperglycemia, and elevated ALT levels, imaging studies that may be useful to assess liver fat include:

• Liver ultrasound is non-invasive and readily available. However, hepatic ultrasound is not sensitive and may miss NAFLD with liver fat content <20% [23,27,32]. Ultrasound has an 80% sensitivity and 86% specificity for detecting moderate to severe hepatic steatosis [3].
• Vibration-controlled transient elastography (VCTE or Fibroscan®) is an ultrasound technique that can measure (a) Controlled Attenuation Parameter (CAP), which is a measure of hepatic steatosis [27] and (b) measure shear wave responses that assess liver stiffness, which infers hepatic fibrosis, inflammation, or congestion [23,33,34]. VCTE machines are portable, noninvasive, and are performed without radiation. However, clinical availably may be limited because the VCTE machine is solely used to evaluate the liver (as opposed to multipurpose MRI scans), and because even when sold used or refurbished, VCTE machines can be cost prohibitive for many providers. VCTE reports often include 2 assessments:
° Controlled Attenuation Parameter (CAP) is an assessment of fat in the liver, ranging from S0 to S3: [3,35].
  ⁃ S0: 0–10% hepatic fat (represents a low CAP score with minimal to no fat in the liver)
  ⁃ S1: 11%–33% hepatic fat
  ⁃ S2: 34%–66% hepatic fat
  ⁃ S3: ≥67% hepatic fat (represents a high CAP score representing severely elevated fat in the liver)
° Fibrosis Score measures hepatic connective tissue that accumulates due to healing from tissue insults such as injury or inflammation. Fibrosis scores vary, depending on the type of liver injury and severity. Fibrosis score may over-estimate fibrosis if the liver has active inflammation, benign tumors, or liver congestion.
  ⁃ F0 – F1: Minimal fibrosis and low liver stiffness
  ⁃ F2: Significant fibrosis some liver stiffness
  ⁃ F3: Severe fibrosis and liver stiffness
  ⁃ F4: Cirrhosis
• Computed tomography (CT) is of limited use for NAFLD due to radiation exposure and limited accuracy in detecting mild hepatic steatosis [32].
• Magnetic resonance imaging-proton density fat fraction (MRI-PDFF) can assess the entire liver, can be used with multiple MRI platforms [27,36], and is a common hepatic imaging procedure performed in NAFLD/NASH drug development programs. As noted, NAFLD assessed by liver biopsy is commonly defined as the presence of ≥5% hepatic steatosis. Conversely, depending on the intent and report, the published and proposed inclusion criteria for fatty liver when assessed by MRI PDFF (proton density fat fraction) may have different proposed cut-off points defining fatty liver, ranging from ≥5.0% to ≥ 12%) [37,38]. [Biomarker Qualification Letter. MRI-PDFF of Liver Tissue as a Diagnostic Enrichment Biomarker https://www.fda.gov/media/124105/download#:∼:text=The%20percentage%20of%20fat%20in,is%20a%20fundamental%20tissue%20property (Submitted 11/2/2018; accessed June 9, 2022)]
• MRI is superior to ultrasound and CT for distinguishing grades of steatosis [3].
• Magnetic resonance spectroscopy (MRS) measures fat in small regions of interest; not all MRI platforms have the capability to perform MRS [32,36].
• Magnetic resonance elastography (MRE) accurately assesses liver fibrosis [39], but is not as available as MRI-PDFF.

### 1.4 NAFLD diagnosis: hepatic fibrosis tools

Hepatic fibrosis tools available for the diagnosis of fatty liver in adults include [23,33]:

• **NAFLD activity score:** histologic diagnosis based upon liver biopsy findings, and involving liver steatosis, lobular inflammation, and liver cell injury ballooning [14].
• **Fibrosis-4 index/calculator:** Is a non-invasive tool that does not involve liver biopsy, and instead incorporates age, AST, ALT, and platelet count [14,40] Fibrosis-4 index (FIB-4) and NAFLD Fibrosis Score (NFS described below) are the two most common noninvasive tools for risk stratification of fibrosis [41]. One meta-analysis suggests that FIB-4 to be associated with a higher performance in ruling in and NFS in ruling out advanced fibrosis [41].
• **NAFLD Fibrosis score:** Estimates fibrosis based upon patient age, body mass index, glucose, AST, ALT, platelet count, and albumin [14].
• **Enhanced Liver Fibrosis score/test:** Incorporates tissue inhibitor of metalloproteinases-1, amino-terminal propeptide of type III procollagen, and hyaluronic acid [14].
• **Fibrometer:** The version for NAFLD incorporates age, weight, platelet count, AST, ALT, ferritin, and glucose [42].
• **FibroSure (United States)/FibroTest (ex-US):** Incorporates age, sex, gamma-glutamyltransferase (GGT), total bilirubin, alpha-2-macroglobulin, apolipoprotein A1, and haptoblobin, as well as ALT when ActiTest is assessed [43].
• **Hepascore:** Incorporates age, sex, and the serum levels of total bilirubin, δ-glutamyl transferase, α2-Macroglobulin, and hyaluronic acid [44,45].

### 1.5 NAFLD diagnosis: assessment

Patients with suspected NAFLD (e.g., those with prediabetes, type 2 diabetes mellitus, pre-obesity/obesity, increase in ALT or AST) may undergo non-invasive screening using the scores previously mentioned, with some preferring the Fibrosis-4 index/calculator [3,14]. Patients found to be at intermediate risk may benefit from VCTE or other fibrosis scores, such as the Enhanced Liver Fibrosis score/test. Patients found to be at high risk or have other lab abnormalities (like elevated autoimmune markers) may benefit from liver biopsy [14]. MRI PDFF and VCTE are often obtained in clinical research trials (and sometimes in clinical practice) to help with diagnosis and monitor the progress of treatment.

### 1.6 NAFLD cause: obesity and adiposopathy

Fatty liver disease most often occurs due to multiple insults in genetically or epigenetically predisposed individuals [46,47]. Genetic causality is supported by family history of NAFLD being a risk factor for NAFLD, as well as identifiable allele variants associated with NAFLD [3]. The ectopic fat deposition in patients with congenital or acquired lipodystrophy is another cause of NAFLD [3]. Among the most common exogenous cause of fatty liver is excessive alcohol consumption. Common secondary disorders that contribute to, or that often accompany NAFLD include: [3,21,23,33,48].

• Adiposopathy with obesity-related immunopathies, endocrinopathies, and the increased circulation of free fatty acids may contribute to “ectopic” deposition of free fatty acids into the liver as well as other body tissues such as muscles, pancreas, and kidneys [[49], [50], [51]].
• Physical inactivity and unhealthful nutrition (e.g., high intake of saturated fats and processed carbohydrates) [52] in genetically susceptible individuals [47,53].
• Type 2 diabetes mellitus
• Insulin resistance
• Components of the metabolic syndrome:
  ✓ Abdominal obesity
  ✓ Hyperglycemia (especially uncontrolled diabetes mellitus)
  ✓ High blood pressure
  ✓ Dyslipidemia (especially hypertriglyceridemia)
• Increase in visceral fat
• Poor sleep & sleep apnea: obstructive sleep apnea (OSA) may increase hepatic fat via promotion of insulin resistance, as well as due to hypoxia, inflammation, endotoxemia, and gut barrier dysfunction.
• Hepatitis (Hepatitis C infection)
• Polycystic ovarian syndrome
• Cardiovascular disease
• Chronic kidney disease

### 1.7 NAFLD cause: concomitant medications

Concomitant medications may contribute to NAFLD and should be reviewed when ​assessing potential causes of NAFLD. These medications include [21,54]:

• Allopurinol (As long as allopurinol does not cause hepatic injury, reducing elevated uric acid with allopurinol may reduce NAFLD [54]).
• Alpha methyldopa
• Amiodarone
• Some antipsychotics
• Some antidepressants
• Aspirin with Reye syndrome
• Corticosteroids (systemic)
• Halothane
• Highly active antiretroviral therapy (HAART)
• Isoniazid
• Lomitapide
• Methotrexate
• Possibly nonsteroidal anti-inflammatory drugs: Data is reportedly inconsistent [55].
• Tamoxifen
• Tetracycline
• Valproate

### 1.8 NAFLD cause: uncommon causes

The following are more uncommon causes of NAFLD [14,21].

• Autoimmune hepatitis
• Celiac disease
• Cholesterol ester storage disease
• Citrin deficiency
• Disorders of lipid metabolism (e.g., abetalipoproteinemia, hypolipoproteinemia, familial combined hyperlipidemia)
• Environmental toxicity, including some industrial solvents
• Glycogen storage disease
• Hypothyroidism [56].
• Lipodystrophy
• Lysosomal acid lipase deficiency (Wolman disease)
• Mauriac syndrome
• Mitochondrial defects in fatty acid oxidation
• Peroxisome dysfunction
• Pregnancy (including HELLP – hemolysis, elevated liver enzymes, low platelet count)
• Reye syndrome
• Starvation and malnutrition
• Surgical rapid weight loss (i.e., bariatric surgery)
• Total parenteral nutrition
• Weber-Christian syndrome
• Wilson's disease

**Fig. 2:**
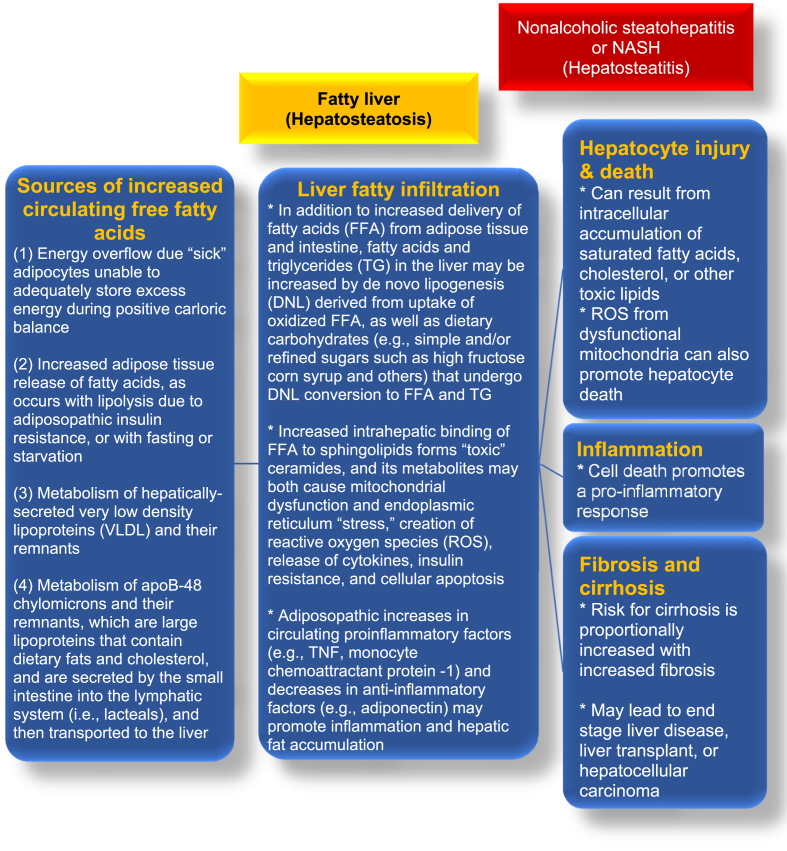
**Free fatty acids and NAFLD.** An adiposopathic increase in free fatty acids can lead to liver fatty infiltration, which in turn may lead to hepatocyte injury/death, inflammation, and fibrosis/cirrhosis [18,[57], [58], [59], [60], [61]].

**Fig. 3:**
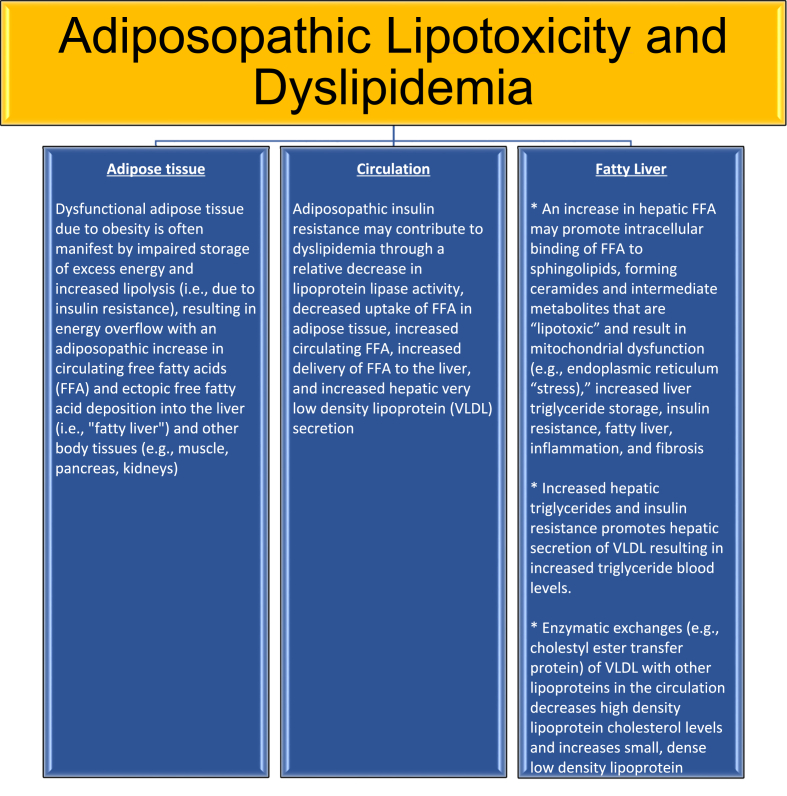
**Lipotoxicity and Dyslipidemia.** Shown are the relationships between the adiposopathic metabolic consequences of obesity, resulting in fatty liver, lipotoxicity, and dyslipidemia [[49], [50], [51],^62^].

One of the primary mechanisms whereby obesity can cause NAFLD is through the adiposopathic increase in free fatty acids (Fig. 2). Increased circulating free fatty acids can lead to hepatic fat deposition, ballooning of hepatocytes, which in turn may lead to hepatocyte injury/death, inflammation, and fibroblast recruitment with end result of fibrosis/cirrhosis [15,16]. Hepatosteatosis or NAFL is fatty liver defined as ≥5% hepatic fat; hepatosteatitis or NASH is fatty liver with inflammation, hepatocyte injury, with or without fibrosis [10,15,16] (Fig. 1). During positive caloric balance, impaired uptake of energy in “sick” peripheral subcutaneous adipose tissue may lead to increased circulating free fatty acids and “ectopic” and pathogenic deposition of free fatty acids into the liver and other body tissues (e.g., muscle, pancreas, kidneys) [49,50]. Fig. 2 shows the relationships between free fatty acids and NAFLD. Fig. 3 shows the relationship of lipotoxicity to fatty liver and dyslipidemia.

### 1.9 NAFLD causes: high fructose corn syrup (versus natural fruit intake)

A fruit is a plant that contains seeds and fiber whose carbohydrate content is often approximately 50% fructose and 50% glucose. Glucose is a simple sugar monosaccharide found in animals and plants with a glycemic index (GI) of 100. Fructose is also a monosaccharide with a glycemic index of GI of 25. The GI of fruit reflect the mixed effect of glucose and fructose, with many citrus fruits having a glycemic index (GI) < 50 [63]. As a frame of reference, table sugar or sucrose (i.e., disaccharide of glucose and fructose derived from sugar cane or sugar beets) has a GI of 65 [64]. Epidemiological data suggest that sucrose and high fructose corn syrup (HFCS), the two most common added sugars to foods and drinks, are not only potentially obesogenic, but also associated with fatty liver, dyslipidemia, insulin resistance, hyperuricemia, cardiovascular disease, type 2 diabetes mellitus, often independent of body weight gain or total energy intake [65].

HFCS is a sweetener originally processed from corn starch, with starch being a chain of glucose molecules used as a plant storage form of carbohydrates [66]. Corn starch is broken down into 100% glucose syrup, with syrup being defined as a liquid containing dissolved sugar. Enzymes are then added to convert some of the glucose into fructose. Fructose taste sweeter than sucrose, with both fructose and sucrose tasting sweeter than glucose. The result is a syrup that is a processed sweetener additive containing higher concentrations of fructose than found in the pure glucose found in corn syrup. Hence the name “high fructose corn syrup.”

Most HFCS is approximately 50% fructose and 50% glucose with a GI of ∼70 [67]. Sucrose (table sugar) is solid and contains covalently bound 50% glucose and 50% fructose with a GI of 65 [67]. While unclear if HFCS and fruit juices (consumed as juice, and not in whole fruit) have the same metabolic consequences, the free sugars content are similar, as are their respective GI [68]. Regarding differences between HFCS and sucrose, HFCS is a liquid originally derived from corn starch, and sucrose is a solid derived from sugar cane and sugar beets. Excessive intake of HFCS and other refined sugars can contribute to obesity, fatty liver disease, hypertriglyceridemia, and diabetes mellitus [67,69,70]. It is challenging to determine the relative pathogenic contributions of the fructose versus glucose components of HFCS when compared to high intakes of other sugars. However, fructose is a potent inducer of lipogenic enzyme expression with the enhanced fatty acid synthesis resulting in increased hepatic diacylglycerols thought to directly interfere with insulin signaling. Fructose may also drive hepatic gluconeogenesis [71].

In contrast to HFCS (i.e., a processed carbohydrate sweetener), natural whole fruit may not be obesogenic [72]. Natural whole fruits with fiber and fructose are more healthful than processed HFCS having no fiber. The clinical consequence regarding the liver is that the HFCS found in candy, processed sweets, soda, fruit juices, and other processed foods is an important cause of NAFLD. HFCS typically has a higher concentration of sugar than most fruit and elicits more rapid intestinal absorption and transport to the liver. Animal studies suggest among the most deleterious macronutrient consumption causing NAFLD is the intake of both saturated fats and liquid fructose [73]. In short, HFCS is a common component of processed carbohydrates, which may be obesogenic and contribute to NAFLD, particularly when accompanied by increased saturated fat intake. Although they contain fructose, consumption of unprocessed natural whole fruit (not fruit juices) is unlikely to be obesogenic and is not thought to substantially contribute to NAFLD [74].

An overall theme is the least healthful macronutrient dietary intake that is most likely to promote NAFLD among patients with obesity includes: [75].

• Sugared drinks (sodas)
• Fruit juices
• Red meat
• Processed meat
• Saturated fats
• Energy dense processed “junk food,” cakes, and biscuits

Dietary intake least likely to promote NAFLD among patients at a healthy body weight includes:

• Whole grains
• Lean meats
• Plant based sources of protein, fruits and vegetables [76].
• Healthful dietary patterns, such as the Mediterranean diet and Dietary Approaches to Stop Hypertension (DASH) diet [77,78].

### 1.10 NAFLD treatment: overview and objectives

Treatments for NAFLD are centered around nutrition, physical activity, and medications. Generally, excessive alcohol intake [e.g., >2 drinks daily (24 oz beer, 8 oz wine, or 2 oz spirits)] is associated with increased risk of alcohol-associated liver disease and cirrhosis. Conversely, moderate ethanol intake (i.e., 1–2 drinks daily) may reduce the risk of NASH and CVD. In patients with established NASH, however, all alcohol consumption should be avoided [3].

Regarding treatment of NAFLD and NASH, the objectives should include: [3].

• Preservation of liver function
• Preventing progression to end-stage liver disease
• Preventing hepatocellular carcinoma
• Preventing metabolic complications (e.g., diabetes mellitus, dyslipidemia, metabolic syndrome), which are cardiovascular disease risk factors

### 1.11 NAFLD treatment: nutrition

For patients with NAFLD, medical nutrition therapy includes an evidenced-based meal plan that helps achieve a healthy body weight, limits saturated and trans fats and ultra-processed/refined carbohydrates [79]. Potential options include the Mediterranean diet with moderate of lean protein (plant or animal based) or other processed carbohydrate/saturated fat restricted nutritional interventions [3,6,77,78]. Among patients with overweight or obesity, weight loss of 3–5% may improve hepatic steatosis with weight loss of 7–10% usually needed to improve histopathological features of NASH (e.g., fibrosis) [[22], [23], [24]].

### 1.12 NAFLD treatment: Dynamic (“aerobic”) and resistance physical activity

Dynamic (aerobic) and resistance-based physical activity help patients achieve and maintain a healthy body weight. Physical activity also increases peripheral insulin sensitivity and reduces circulating free fatty acids and glucose, which reduces their delivery to the liver [80,81]. Lastly, physical activity increases intrahepatic fatty acid oxidation, decreases fatty acid synthesis, and helps prevent mitochondrial and hepatocellular damage [23,80], which may have therapeutic benefits in treating NAFLD, with favorable effects that may be independent of weight loss [3].

### 1.13 NAFLD treatment: weight reduction in patients with pre-obesity/obesity

Among patients with pre-obesity/obesity, weight reduction of ≥10% achieved by healthful nutrition (i.e., Mediterranean diet versus low fat diet) and enhanced energy expenditure (i.e., routine physical activity) can potentially improve NAFLD and NASH over a relatively short period of time [3].

### 1.14 NAFLD treatment: medications

No pharmacotherapy has an approved indication to treat NAFLD [8]. Despite intuitive expectations, medications such as metformin and dipeptidyl peptidase IV inhibitors have not been proven to reduce liver fat or treat NASH [14]. Among pharmacotherapy that may help treat NAFLD and its complications include:

• Vitamin E 800 IU may provide biochemical and histological improvement in fatty liver in some adult patients with NASH without diabetes mellitus and without cirrhosis [3,8,82]. However long-term use may increase rates of prostate cancer [83].
• Peroxisome proliferator activated receptor gamma agonists (i.e., pioglitazone) may reduce liver fat and improve NASH [14,23,24], even as body weight/fat may be increased [3].
• Glucagon-like protein–1 receptor agonists may reduce liver fat and improve NASH [14,23,24]. Some evidence suggests liraglutide may improve NAFLD [3]. In a study of 320 patients with biopsy-confirmed NASH and liver fibrosis, treatment with semaglutide resulted in greater improvement in NASH resolution than placebo but did not demonstrate a difference in the percent of patients with an improvement in fibrosis stage [84].
• Leptin therapy in patients with lipodystrophy may improve NAFLD [3].

### 1.15 NAFLD treatment: bariatric surgery

Bariatric surgery may not only improve type 2 diabetes mellitus, dyslipidemia, and hypertension, and reduce cardiovascular morbidity and/or mortality [85], but it may improve liver histology including fibrosis secondary to NASH [26].

## 2 Conclusions

This OMA CPS on nonalcoholic fatty liver disease and obesity is one of a series of OMA Clinical Practice Statements designed to assist clinicians in the care of patients with the disease of obesity. Knowledge of the relationship between obesity and nonalcoholic fatty liver disease, inclusive of diagnosis and treatment, may help improve the care of patients with pre-obesity/obesity, particularly those patients with adverse fat mass and adiposopathic metabolic consequences.

## Transparency [86]

This manuscript was largely derived and edited from the 2021 Obesity Medicine Association (OMA) Obesity Algorithm. Beginning in 2013, OMA created and maintained an online Adult “Obesity Algorithm” (i.e., educational slides and eBook) that underwent yearly updates by OMA authors and was reviewed and approved annually by the OMA Board of Trustees. This was followed by a similar Pediatric “Obesity Algorithm,” with updates approximately every two years by OMA authors. Authors of prior years’ version of the Obesity Algorithm are included in Supplement #1.

## Group composition

Over the years, the authors of the OMA Obesity Algorithm have represented a diverse range of clinicians, allied health professionals, clinical researchers, and academicians. (Supplement #1) The authors reflect a multidisciplinary and balanced group of experts in obesity science, patient evaluation, and clinical treatment.

## Author contributions

SK, AA, TF, and HEB reviewed, edited, and approved the document.

## Managing disclosures and dualities of interest

Potential dualities or conflicts of interest of the authors are listed in the Individual Disclosure section. Assistance of a medical writer paid by the Obesity Medicine Association is noted in the Acknowledgements section. Neither the prior OMA Obesity Algorithms, nor the publishing of this Clinical Practice Statement received outside funding. The authors of prior OMA Obesity Algorithms never received payment for their writing, editing, and publishing work. Authors of this Clinical Practice Statement likewise received no payment for their writing, editing, and publishing work. While listed journal Editors received payment for their roles as Editors, they did not receive payment for their participation as authors.

## Individual disclosures

SK reports to be a member of the Speaker's Bureau for Abbott Nutrition. AA is a member of the speaker's bureau for NovoNordisk, Vivus, and Currax. He also serves as an advisor to Néstle Health Science and Phenomix Sciences. HEB reports his research site institution has received research grants from 89Bio, Alon Medtech/Epitomee, Altimmune, Amgen, AstraZeneca, Boehringer Ingelheim, Eli Lilly, Madrigal, NovoNordisk, and Pfizer. HEB reports being a consultant for 89Bio, Amgen, Altimmune, and Boehringer Ingelheim. TF reports her employer as the Obesity Medicine Association, but no further disclosures.

## Evidence

The content of the OMA Obesity Algorithm and this manuscript is supported by citations, which are listed in the References section.

## Ethics review

This OMA Clinical Practice Statement manuscript was peer-reviewed and approved by the OMA Board of Trustee members prior to publication. Edits were made in response to reviewer comments and the final revised manuscript was approved by all the authors prior to publication. This submission did not involve human test subjects or volunteers.

## Conclusions and recommendations

This Clinical Practice Statement is intended to be an educational tool that incorporates the current medical science and the clinical experiences of obesity specialists. The intent is to better facilitate and improve the clinical care and management of patients with pre-obesity and obesity. This Clinical Practice Statement should not be interpreted as “rules” and/or directives regarding the medical care of an individual patient. The decision regarding the optimal care of the patient with pre-obesity and obesity is best reliant upon a patient-centered approach, managed by the clinician tasked with directing an individual treatment plan that is in the best interest of the individual patient.

## Updating

It is anticipated that sections of this Clinical Practice Statement may require future updates. The timing of such an update will depend on decisions made by Obesity Pillars Editorial team, with input from the OMA members and OMA Board of Trustees.

## Disclaimer and limitations

Both the OMA Obesity Algorithms and this Clinical Practice Statement were developed to assist health care professionals in providing care for patients with pre-obesity and obesity based upon the best available evidence. In areas regarding inconclusive or insufficient scientific evidence, the authors used their professional judgment. This Clinical Practice Statement is intended to represent the state of obesity medicine at the time of publication. Thus, this Clinical Practice Statement is not a substitute for maintaining awareness of emerging new science. Finally, decisions by practitioners to apply the principles in this Clinical Practice Statement are best made by considering local resources, individual patient circumstances, patient agreement, and knowledge of federal, state, and local laws and guidance.
